# Mapping respiratory health digital interventions in South and Southeast Asia: a scoping review

**DOI:** 10.7189/jogh.15.04310

**Published:** 2025-11-14

**Authors:** Laura Evans, Jay Evans, Paul Barach, Adina Abdullah, Zakiuddin Ahmed, RESPIRE Collaboration

**Affiliations:** 1 Usher Institute of Population Health Sciences and Informatics, The University of Edinburgh, Edinburgh, UK; 2 Thomas Jefferson University, Philadelphia, USA; 3 Sheps Health Services Research Center, University of North Carolina, Chapel Hill, USA; 4 Imperial College London, London, UK; 5 Department of Primary Care Medicine, Universiti Malaya, Kuala Lumpur, Malaysia; 6 Riphah Institute of Healthcare Improvement & Safety, Riphah International University, Islamabad, Pakistan

## Abstract

**Background:**

The growing burden of respiratory disease, particularly in Asia, where mortality is higher and awareness and policy engagement lag, could be mitigated through rapidly advancing digital health tools that offer opportunities for improved management, prevention, and personal health empowerment. We aimed to map the existing evidence, technologies, opportunities, and gaps related to respiratory digital health interventions in South and Southeast Asia and propose relevant recommendations.

**Methods:**

We used a scoping review methodology, where we searched MEDLINE, Embase, CINAHL, PsycINFO, Cochrane Library, Web of Science, PakMediNet, and MyMedR along with grey literature databases (ProQuest Thesis and Dissertations, Digital Health Atlas, Global Digital Health Monitor, Global Index Medicus) for reports on any technological interventions for pneumonia, tuberculosis, asthma, chronic obstructive pulmonary disease, and environmentally induced respiratory disease (air quality, smoking). We used the World Health Organization’s Classification of Digital Health Interventions to categorise digital interventions and assessed how completely they were reported *via* the mHealth Evidence Reporting and Assessment checklist.

**Results:**

We extracted and analysed data from 87 studies conducted in 14 South and Southeast Asian countries and found that digital health interventions are primarily used for communication with patients and between patients and providers. Interventions targeting tuberculosis were the most numerous. There was a high prevalence of pilot interventions which failed to significantly address the respiratory health needs in the region. Artificial intelligence and machine learning interventions are promising, but lack clear guidelines and adherence to best ethical and equity practices.

**Conclusions:**

We collated and synthesised information and knowledge about the current state of digital health interventions. Our findings can inform future interventions so that they are planned, deployed, scaled, and evaluated to have long-lasting positive impacts on population health.

**Registration:**

Evans L, Evans J, Fletcher M, Abdullah A, Ahmed Z. Mapping Respiratory Health Digital Interventions in South and Southeast Asia: Protocol for a Scoping Review. 2024;13:e52517.

The burden of respiratory disease is a major concern worldwide, with both infectious and non-communicable respiratory diseases contributing to increased morbidity and mortality [1,2]. The burden of respiratory disease is particularly significant in low- and middle-income countries (LMICs), where mortality rates are higher, and public awareness and government engagement are lower than in other regions of the world [3,4]. This reality is aggravated by the ‘second translational gap’, whereby respiratory health research findings fail to find a way into clinical practice and a positive personal health impact, especially in LMICs [4,5]. Digital health interventions (DHIs) can be leveraged to help bridge this gap [6].

Digital health, defined as the use of information communication technologies to manage patient illnesses and wellness, has progressed rapidly due to advances in technology and a promise of improved health outcomes, increased health system efficiencies, greater health equity, and personal health empowerment [7,8]. The COVID-19 pandemic and the rapid spread of artificial intelligence (AI) technology have exponentially increased the pace of growth and relevance of DHIs [9]. However, indiscriminate development and deployment of DHIs is unhelpful and potentially harmful in that it uses valuable human, logistical, and economic resources without meaningful and measurable population health results [10]. Such harm is exacerbated in LMICs, where resources are more scarce and highly dependent on external players (such as funders, neighbouring countries, rapid political turnover, *etc*.) [10]. Therefore, DHIs need to be planned carefully, with timely assessments of their potential impact on population health and health systems.

A critical step in harnessing the power of digital health requires understanding the current digital landscape. Knowing what current DHIs are being deployed and used effectively (or not) may help reduce costs, avoid duplication, and increase the efficiency, accessibility, and sustainability of interventions [8–11]. Several reviews (particularly scoping reviews) have analysed different aspects of DHIs [11–15]; however, to our knowledge, none have focussed on respiratory health in South and Southeast Asia. Our aim was to map respiratory DHIs in these contexts, identifying existing technologies, opportunities, and gaps, and putting forward pertinent recommendations. Our research questions were:

1. What digital health tools and technologies are being employed in South and Southeast Asia for respiratory health?

2. How are these tools addressing (or not) the respiratory health needs of the region?

3. What recommendations can be made from the literature?

## METHODS

### Scoping review methodology

We used the scoping review methodology proposed by Arksey and O’Malley and the Joanna Briggs Institute [16,17], and reported our findings using the PRISMA extension checklist for scoping reviews (PRISMA-ScR) [18] to ensure adherence to methodological standards (**Online Supplementary Documents**). Specific details on the methods for this study can be found in the published protocol [19].

### Data sources and search strategy

We searched MEDLINE, Embase, CINAHL, PsycINFO, Cochrane Library, Web of Science, PakMediNet, and MyMedR, as well as grey literature databases (ProQuest Thesis and Dissertations, Digital Health Atlas, Global Digital Health Monitor, Global Index Medicus). The search strategy was iteratively developed and refined by the authors’ input and the librarian at the University of Edinburgh. The terms ‘respiratory health’, ‘digital health’, ‘South Asia’, ‘Southeast Asia’, and all relevant variations of these terms were included in the search strategy to gather as much pertinent literature and information as possible (**Online Supplementary Document**).

### Study selection

We developed our study inclusion and exclusion criteria according to the ‘population’, ‘concept’, ‘context’, and ‘type of evidence’ domains suggested by the Joanna Briggs Institute [18,20]. We further created an ‘other variables’ category to include the year of publication, language, and format criteria (**Table 1**).

**Table 1:** Inclusion and exclusion criteria

Domain	Inclusion criteria	Exclusion criteria
Population	Population of any age and background within the regional focus	N/A
Concept	Technological interventions for respiratory health that fall under any of the categories of the WHO classification of digital health interventions, with respiratory health including respiratory infections, non-communicable respiratory diseases, and preventable risk factors for respiratory conditions as defined by RESPIRE	Other non-technological interventions used for respiratory health, or lacking a respiratory health focus
Context	South and Southeast Asia	Rest of the world
Types of evidence sources	Peer review and grey literature publication	Books, abstracts, posters, protocols
Other variables	Published in English, studies or data published in the last 10 years (2013–23), full article/data available digitally	Published in any other language, studies or data published before 2013, full article/data not available digitally

NA – not applicable, RESPIRE – Research Unit on Respiratory Health, WHO – World Health Organization

We included studies from 19 countries belonging to the regions of South and Southeast Asia, as defined by the United Nations Statistics Division [21]. We also included multi-country studies in countries from the selected regions and other regions of the world in the initial screening and only excluded them if they did not provide the data of interest separately for each country. Only studies in English were included, as that was the common language for all researchers in our team. We included publications from 2013 to July 2023 (when the study started), as technology older than ten years is likely irrelevant due to disuse or upgrades. The databases were searched and the results imported into Covidence in July 2023, while the screening and data extraction took place between September 2023 and June 2024, as the researchers’ availability allowed.

We used the Covidence software [22] to eliminate duplicates and perform screening. After deduplication, two authors (LE and JE) performed the title, abstract, and full-text screening, discussing any discrepancies and, if needed, consulting a third reviewer (AA).

One reviewer (LE) screened the reference lists of included studies for additional relevant studies. She entered relevant data into a spreadsheet, where it was assessed by a second reviewer. Discrepancies were addressed discussed between both reviewers. The references of eligible studies were manually checked to identify additional relevant studies that were missed in the database searches.

Our scoping review protocol included COVID-19 as one of the diseases in the search criteria. However, when the screening started, we decided to exclude studies that focussed exclusively on COVID-19, as we encountered many studies involving other health issues besides respiratory symptomatology, such as inflammation, headaches, fatigue, or long COVID [23]. Therefore, it became time-consuming and challenging to screen for studies that only looked at respiratory symptoms related to COVID-19, as many studies did not state this explicitly. Additionally, the DHIs developed and deployed during COVID-19 had a very specific purpose, as in previous large-scale outbreaks [24], and were at risk of being abandoned or decommissioned once the pandemic ended. We did not assess the included studies for quality beyond the inclusion criteria, as per scoping review guidelines [20]. 

### Data extraction and analysis

After the screening process, we extracted the relevant data and entered them into a spreadsheet which we had previously piloted in five studies. We first presented quantitative data (*i.e.* the number of studies, type of study, and year), after which we inductively analysed qualitative data (description, aim, results of the interventions) for emerging themes and presented them *via* a narrative synthesis.

For deductive analysis, we used the WHO Classification of Digital Health Interventions [25]. This classification categorises ‘the different ways in which digital and mobile technologies are being used (use cases) to support individuals and health system needs’ and ‘acknowledges the diverse ways in which (DHIs) are leveraged in different applications and services, to address personal and health system challenges and needs’ [25]. The four main categories in this classification are ‘DHIs for persons’ (for individuals and/or patients to use), ‘DHIs for healthcare providers’, ‘DHIs for health managers and support personnel’, and ‘DHIs for data services’ (to collect, manage, analyse, and visualise data). These, in turn, are subdivided into further categories.

Additionally, we utilised the mHealth Evidence Reporting and Assessment Checklist (mERA) [26] to report on the comprehensiveness of reporting on the DHIs interventions. The mERA checklist aims to identify a minimum set of information needed to define a mHealth intervention (content), where it is being implemented (context), and how it was implemented (technical features), to support replication of the intervention [26]. We selected this standardisation checklist over others, such as CONSORT-EHEALTH [27], because of its global focus and the comprehensiveness of its domains. 

### Consultation

Consultations with academic and clinical partners in the RESPIRE consortium [28] were ongoing throughout the scoping review process. We disseminated early findings among RESPIRE partners *via* a poster presentation, and inviting feedback on findings to be incorporated into the discussion.

### Ethical considerations

Scoping reviews use secondary data and do not require ethics or institutional approvals under RESPIRE rules. This methodology established a transparent and reproducible study design, which limits the potential for personal bias [19].

## RESULTS

**Figure 1:**
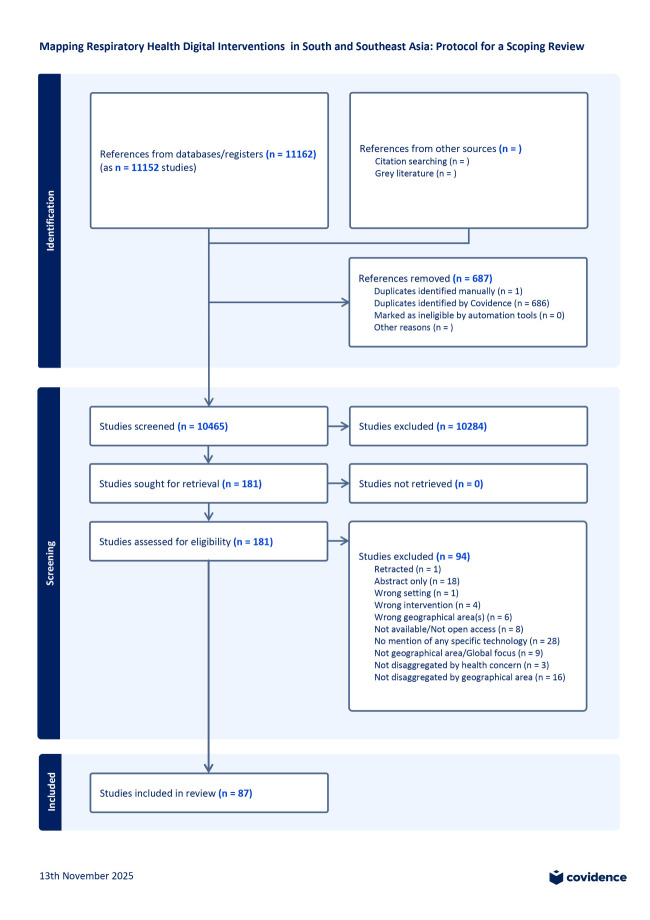
PRISMA-ScR flowchart for this scoping review [18].

The database searches yielded 11152 studies that were imported into Covidence (**Figure 1**). Of those, 687 were removed during deduplication; 10 465 study titles were subsequently screened, and 10 285 were found irrelevant and excluded. We screened the abstracts and full texts of 181 studies, excluding 94 studies for various reasons retaining 87 for analysis. Three of the remaining studies were reported in more than one publication and were combined into one for analysis purposes.

**Figure 2:**
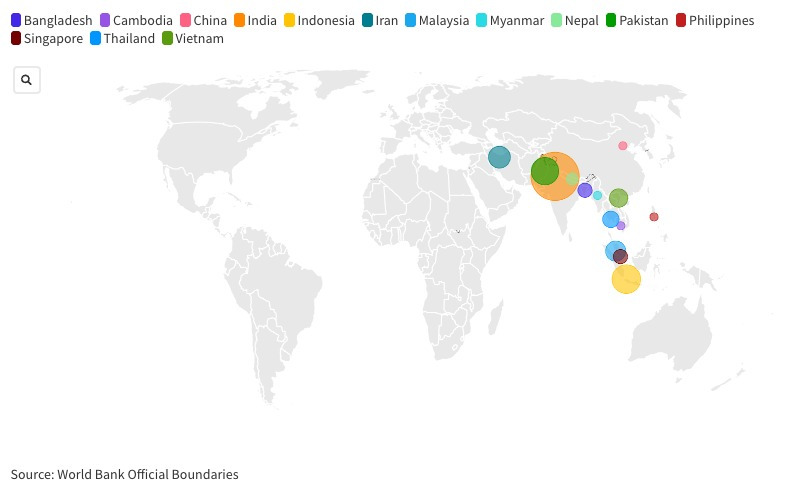
Studies took place in the following countries: India (n = 34), Indonesia (n = 12), Pakistan (n = 10), Iran (n = 7), Malaysia (n = 6), Vietnam (n = 5), Thailand (n = 4), Bangladesh (n = 3), Nepal (n = 2), Singapore (n = 3), Cambodia (n = 1), China (n = 1), Myanmar (n = 1), and Philippines (n = 1).

The 87 studies were conducted in 14 South and Southeast Asian countries (**Figure 2**; **Online Supplementary Document**). The largest number was done in India (n = 34), followed by Indonesia (n = 12) and Pakistan (n = 10). Three studies involved two or more countries.

The number of relevant studies published grew significantly from 2019 onwards compared to years prior. Specifically, one or two studies were published annually between 2013 and 2016, followed by an increase to five studies per year during 2017 and 2018. Starting in 2019 up to 2023, 11 to 14 relevant studies were published each year, except in 2021, when 20 such studies were published.

**Figure 3:**
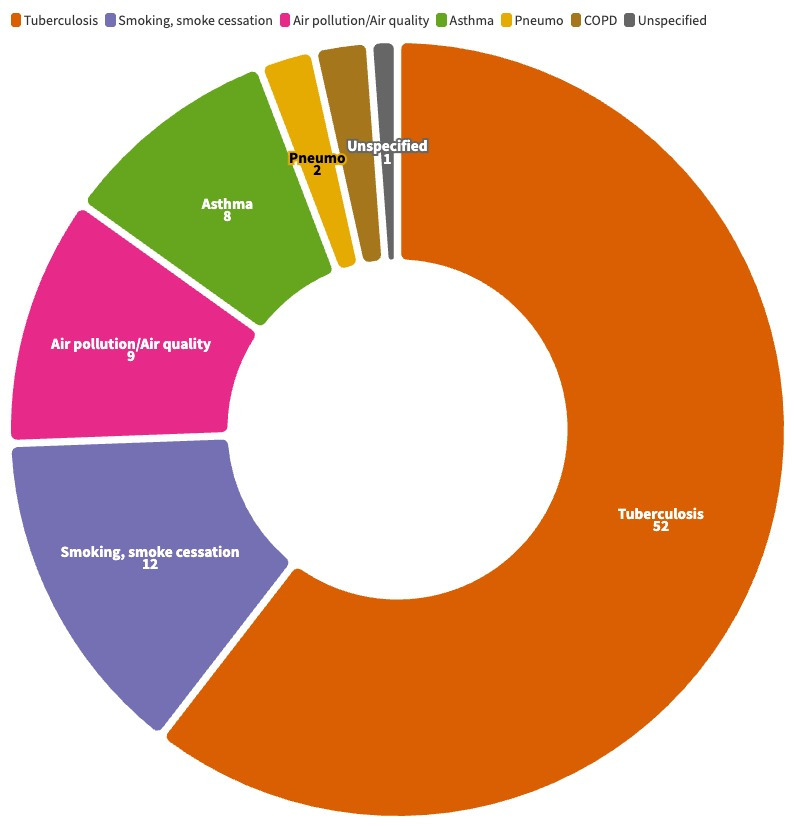
Number of studies by disease/health concern.

In terms of health concerns, most studies (n = 52) focussed on tuberculosis (TB), followed by smoking cessation interventions (n = 12), air quality and air pollution interventions (n = 9), asthma (n = 8), chronic obstructive pulmonary disease (n = 2), and pneumonia (n = 2). One study focussed on lung disease in general (**Figure 3**).

### Early-stage DHIs

Of the 86 studies, 71 were prototypes or pilots. Prototypes frequently refer to DHIs with limited functionality, and are created to assess their usability, feasibility, or acceptability [29]. Pilots can be defined as specific technologies deployed in particular settings for a limited time to assess their effectiveness, safety, and cost-saving potential [30]. We observed two different types of prototypes and pilots in the included literature. The first were studies that developed a prototype or a pilot DHI from scratch, *i.e.* created a dedicated piece of technology to carry out the study, with no further intention of scale-up beyond the study, or at least with no such intention expressed in the related report. The second were studies that, although were designated as pilot projects according to the above definition, used already existing and, at many times, well-documented technological tools. These were primarily studies using SMS, phone calls, Computer-Aided Detection for Tuberculosis (CAD4TB), Directly Observed Treatment, Short Course (99DOTS), and ArcGIS Geographic Information System.

### Criteria of the WHO Classification of Digital Health Interventions

When looking at the included studies, we see that the most frequently reported use cases for TB are ‘DHIs for healthcare providers’ and ‘DHIs for persons’. For smoking cessation and for asthma, the most commonly utilised use case is ‘DHIs for persons’, while for air quality and air pollution, ‘DHIs for data services’ is the most prevalent use case (**Table 2**). We note, however, that a single DHI may have multiple use cases.

**Table 2:** WHO’s Classification of DHIs’ number of times use cases by disease/health concern

Condition	DHIs for persons	DHIs for health care providers	DHIs for health managers	DHIs for data services
Tuberculosis	23	27	2	6
Smoking, smoking cessation	11	1	0	1
Air quality/air pollution	2	0	0	8
Asthma	5	1	0	3
Pneumonia	0	1	0	1
COPD	1	1	0	0

COPD – chronic obstructive pulmonary disorder, DHI – digital health intervention

### mERA checklist assessment

Studies most frequently reported on the technology platform used, the way the intervention was delivered, the content of the intervention and how people were engaged and adopted the intervention. The least reported aspects were some of the more critical ones, such as interoperability, cost assessment, data security and regulation (**Table 3**).

**Table 3:** Number of studies reporting on different aspects of the intervention, classified according to the mERA checklist

mERA specification	Short description	Number of studies reporting aspect of intervention
Technology platform	Describes the technology architecture, including software and hardware details	81
Intervention delivery	Describes the mode of delivery of intervention (that is, SMS, face to face, interactive voice response)	55
Adoption inputs/programme entry	Describes how people are informed about the programme including training required to implement the DHI	47
Intervention content	Details of the content of the intervention are described	41
Access of individual participants	Mentions barriers or facilitators to the adoption of the intervention among study participants, including structural, economic and social barriers or facilitators	35
User feedback	Describes user feedback about the intervention or user satisfaction with the intervention	31
Usability/content testing	Describes formative research and/or content and/or usability testing with target group(s) clearly identified	24
Fidelity of intervention	Describe the strategies employed to assess the fidelity of the intervention	22
Limitations for delivery at scale	Clearly presents the DHI’s limitations for delivery at scale	14
Contextual adaptability	Describes the adaptation, or not, of the solution to a different language, different population or context	14
Infrastructure	Clearly presents the availability of infrastructure to support technology operations in the study location	10
Replicability	Presents the source code/screenshots/ flowcharts of the algorithms or examples of messages to support replicability of the DHI in another setting	9
Data security	Describes the data security procedures/ confidentiality protocols	5
Compliance with national guidelines or regulatory statutes	Mechanism used to assure that content or other guidance/information provided by the intervention is in alignment with existing national/regulatory guidelines	5
Interoperability/HIS context	Describes how mHealth intervention can integrate into existing health information systems/digital health infrastructure	4
Cost assessment	Reporting of some cost considerations for the DHI in lieu of a full economic analysis	4

DHI – digital health intervention, mERA – mHealth Evidence Reporting and Assessment

### Old technologies reimagined

We found a wide range of technologies used in the studies. This included, for example, older technologies, such as short message service (SMS) and phone calls, which are still valid and effective in many settings due to their low cost and familiarity with the technology [31]. However, some of the studies [32,33] used what can be considered a new iteration of the SMS – WhatsApp – to transmit targeted messaging to patients the same way it would have been done through SMS. Moreover, some studies present yet another iteration, which is an upward trend, of the two-way messaging system: using AI-powered chatbots [34–36]. The studies used these kinds of chatbots specifically for capacity building for health workers in the prevention and management of TB.

Another old technology being ‘reimagined’ is the x-ray. Ten studies evaluated the accuracy of machine learning (ML) and AI technologies applied to chest x-rays (CXR) in patients with TB. The majority studied the CAD4TB software [37–47], which can quickly interpret a CXR image using deep-learning technology and is designed for patients older than four, and is already being used in various settings with good performance [48]. All of the studies found applying AI and ML to CXR interpretation to be proper and efficient. The countries represented in this study have low economic resources, which impact the availability of Genexpert (the gold standard for TB diagnosis), and limited human resources, which affects the possibility of qualified radiologists interpreting CXRs quickly. Therefore, measuring the utility and accuracy of interventions like CAD4TB is sensible. 

### AI and ML

Beyond being used to screen patients by risk or health status (such as in the case of CAD4TB), AI and ML were employed for automated data analysis to generate new information or predictions on future events related to air quality and air pollution, tobacco use, TB, and pneumonia [49–63]. Although these studies found AI and ML beneficial and highly accurate, all were cautious about endorsing them at a large scale, recommending more extensive trials and research before scaling up.

The included studies demonstrate that AI and ML can have safe and effective real-life applications when integrated into clinical decision-support tools [23,24,50,62,64]. They can also be used as public health tools for predicting and forecasting adverse health effects, such as increased air pollution or disease surveillance tools for infectious diseases such as TB or pneumonia.

## DISCUSSION

We aimed to map the respiratory DHIs in South and Southeast Asia to identify existing technologies, opportunities, and gaps and subsequently put forward pertinent recommendations. Our scoping review findings provide a preliminary snapshot of existing digital health tools in South and Southeast Asia being used for respiratory health. We found that the number of DHIs employed matches the prevalence of diseases in the region, that most of the studies report on prototypes or pilots and not mature interventions, and that AI and ML are becoming highly relevant players in digital health.

### DHIs and respiratory health

Although we answered the first question of this scoping review (What digital health tools and technologies are being employed in South and Southeast Asia for respiratory health?) when presenting our results above, we discuss other relevant insights here as well. The high number of studies investigating the use of DHIs for TB (DHIs are employed in all four use cases detailed in the WHO’s Classification), which is aligned with the high prevalence of TB in the region, demonstrates an interest from national and international stakeholders, translating into funding to find digitally-enabled effective ways to diagnose, treat, and cure TB. While this is welcomed by researchers, implementers, clinicians, and patients, it is worth highlighting that, to achieve the greatest impact at the population and health system levels, DHIs should be inclusively co-created with all relevant stakeholders, including through discussions of how available funding may be best used [65]. This holds true for DHIs across all respiratory diseases/health concerns identified in this scoping review, and it is in line with other existing literature and guidelines such as the Principles for Digital Development [66], the WHO’s Digital Health Strategy [67] and the Asian eHealth Information Network [68].

A notable gap uncovered in our analysis lies in the limited focus on chronic and environmentally influenced respiratory diseases (asthma, COPD, and air pollution/air quality). Addressing this imbalance is essential to ensure that digital health innovations benefit the full spectrum of respiratory conditions, not only acute or well-funded areas. Asthma, COPD, and respiratory diseases due to environmental factors pose a significant burden of disease long-term (even if lower than TB, for example), yet are being deprioritised from a funding and intervention perspectives compared to other diseases such as TB, which may offer short-term ‘wins’ for funders and practitioners [69].

Another insight of this analysis is that none of the DHIs presented in the studies were systematically evaluated by the authors of the studies. No study used a standardised checklist or framework (mERA or any other) to report on the DHI. Most studies, however, describe and report on the intervention presented at length and in detail. Nevertheless, utilising standardised guidelines to describe and evaluate DHIs when reporting results can add significant value by increasing the quality and comparability factors across DHIs. Moreover, when guidelines are utilised from the beginning of a project, it can help guide the design, development, deployment, and evaluation of DHIs, making researchers and implementers aware of all the critical aspects of these interventions.

### To prototype or not to prototype?

To answer our second research question (How are the current DHIs addressing (or not) the respiratory health needs of the region?), we addressed the high prevalence of pilots and prototypes in the included studies. Almost 82% (n = 71) of the studies involved a DHI that was a prototype or a pilot. These studies, overall, stated favourable results, and yet very few reported on key mERA categories for scale-up, such as replicability at scale, data security, compliance with national guidelines, interoperability, or cost assessment, a likely indicator that no concrete plans for the growth of the intervention existed at the moment of reporting. Much insight can be gained from early-stage DHI projects, such as the appropriateness of the tool, the acceptability, usability, functionality, *etc*.; in fact, prototypes and pilots are a necessary part of deploying DHIs. However, they cannot have an impact at the population level [70–72] and, therefore, cannot address the respiratory issues faced by whole communities.

We offer two potential approaches to this question so that progress can be made regarding DHIs meaningfully addressing the respiratory needs of whole communities. First, we recommend focussing on sustaining innovations. Christensen and colleagues [73] talk about disruptive and sustaining innovations; the former reinvent a technology or simply invent something new altogether, while the latter improve existing products and processes. Many of the prototypes and pilots concentrated on creating a new piece of technology to address a specific respiratory health issue, which could be classified as an attempt to develop a disruptive innovation. However, focussing on repurposing, improving, or adapting existing technologies for specific contexts and respiratory health issues (a sustaining innovation) may be a better approach and one that has been used effectively before [74]. For example, some of the studies used existing digital health tools and adapted them to their context or, as in the case of CAD4TB, evaluated them with local data sets to understand their performance. This can add significant value because it uses a well-established, well-functioning tool and evaluates it in a specific context. If the evaluation is favourable, the speed at which that tool – in this case CAD4TB – can be upscaled is much greater than a tool built from scratch because of its proven effectiveness and safety track record.

Yet, this is often not enough. Besides focussing more on studying and trialling sustaining innovations, a systematic and rigorous approach to early-stage DHI projects is paramount. Most of the included studies sought to evaluate narrow specifications (feasibility, usability, accuracy, reliability, *etc*.) instead of having a holistic perspective. When using the mERA checklist to understand what aspects of their DHIs authors reported on, we noted that several aspects were critically underreported. Failing to evaluate and report the key factors that influence a DHI can create a distorted understanding of the potential an intervention has to scale and a potential society-level impact. The characteristics of a prototype or a pilot are limited in terms of place (deployed in one place or just a few) and time (for a month, six months, or other limited amount of time). Beyond this, for a prototype or a pilot to significantly fulfil its purpose - that is, its effectiveness – safety and cost-saving potential, it must encompass many aspects of a real, large-scale implementation. Using standardised checklists and toolkits such as mERA, a systematic procedure for developing, evaluating and reporting results, can be applied here, and would allow to better appreciate what is required to scale up a particular DHI, what resources may be needed, and what critical issues may arise on the way.

### Case study: Nikshay and 99DOTS

At the other end of the spectrum from pilot projects, we found the Nikshay and 99DOTS interventions. Nikshay ‘(Ni = End, Kshay = TB) is an Indian web-based patient management system for TB control under the National Tuberculosis Elimination Programme (NTEP)’ [75], while 99DOTS ‘is a low-cost approach for monitoring and improving TB medication adherence, (that) enables remote observation of doses administered by patients or their family members’ [76] Several of the included studies deal with different aspects of these two programmes [77–84], evaluating the accuracy, acceptability, feasibility, data management, and other aspects, but all within the context of these mature interventions.

99DOTS is now part of Nikshay, reflecting a greater sustainability as proven interventions merge to simplify work processes for patients and providers. There are key things that can be learned from the Nikshay intervention. First, it is a fitting example that institutionalisation in the form of government funding and support can have a critical impact at scale and that integration with other interventions through national data standardisation processes can simplify workflows [67,85]. Second, it reflects the reality that flexible and ‘old’ technology deployed in resource-constrained and signal-poor areas can scale up in a sustainable manner. Third, conscientious capacity-building efforts were built into the Nikshay and 99DOTS interventions, ensuring through the whole lifecycle of the interventions that the health care workers who use these tools also have access to training. Finally, the Nikshay example shows how national-level policy is critical to support the development of DHIs [72,86,87].

The success of Nikshay does not imply that the intervention is perfect nor that it does not face sustainability challenges. A report from Stop TB Partnerships highlights several areas for improvement, such as the need to adopt international interoperability standards, add real-time data functionality, eliminate duplicate, paper-based tasks, and expand infrastructure and capacity-building endeavours [87]. Knowing the successes, failures, and areas of improvement of interventions such as with Nikshay and 99DOTS as they grow and scale over time allows implementers and researchers to distil lessons learnt that can positively impact the development and deployment of future interventions. Moreover, the studies included here that look at the different aspects of Nikshay and 99DOTS are effectively sustaining innovations that seek to improve and expand upon a functioning and established intervention, potentially increasing their impact.

### AI and ML

Interventions using AI and ML were abundant in this review and had positive accuracy results. However, the authors were cautious about endorsing such technologies without more evaluation studies, suggesting that more research is needed with larger data sets or in different settings.

The DHIs in the regions of South and Southeast Asia can capitalise on AI and ML technologies early on, as they deploy DHIs that are AI/ML ready instead of adopting legacy systems from other countries with more mature digital health ecosystems [88]. Some early best practices around AI are already emerging. These are predominantly focussed on the ethical use of AI, preventing bias in AI and ML algorithms, considering health equity, utilising explainable AI (and not relying on the ‘black box’ explanation), stringent data privacy regulations, and sufficiently large training data sets that match real-life data [89]. Such practices and recommendations need to be carefully heeded so that the region can maximise the benefits of AI and ML while minimising the risk to LMIC citizenry.

There is growing fear that AI and ML will erase healthcare jobs in the coming years, and although some administrative tasks may be automated in the future [90], the disappearance of entire roles is unlikely to occur [91]. Instead, their primary purpose is (and likely will be) to augment and enhance human skills and knowledge and facilitate faster, more accurate decision-making processes [92]. The potential of AI and ML for air quality prediction, TB diagnosis, respiratory infectious disease incidence and transmission forecasting, and clinical decision support systems is hard to overestimate. However, their full potential will not be realised without the human ‘touch’ [93] and if ethical, equitable, and transparent policies, practices, and evaluation mechanisms for AI and ML are firmly established in the region.

### Recommendations

We propose three main recommendations based on the findings of our scoping review for those involved in the planning, design, development, deployment, and management of DHIs for promoting respiratory health quality in the region, thereby answering our third research question (‘What recommendations can be made from the literature?’).

#### Recommendation 1: assess the need for another pilot project

Prototypes and pilots are key pathways to test new concepts, but have demonstrated limited sustainability, impact, and integration into existing systems and workflows. Therefore, before initiating a new project, thoroughly evaluate the need for a prototype or a pilot project and justify it. A rigorous needs assessment and justification process should be undertaken that considers micro- and macro-level factors. Consider whether building on what already exists is a better option for sustainability and impact. Utilising and iterating established digital health tools can better support interoperability, reduce fragmentation, and increase the opportunity for long-term sustainability.

#### Recommendation 2: report on interventions systematically

Incomplete or inconsistent reporting hampers the ability to meaningfully compare outcomes, distil learnt lessons, and inform policy. Before starting the project, decide on developmental, formative and summative evaluation metrics and parameters used for reporting. Many existing frameworks can aid this process. While we utilised the mERA checklist in this scoping review, other tools can also be used, depending on needs. Deciding beforehand will help in choosing metrics, enabling systematic reporting, reproducibility, and knowledge building, thereby increasing the reliability of the project and allowing the entire digital health community to learn more valuable lessons.

#### Recommendation 3: ensure the responsible adoption of AI and ML

AI and ML deployment are rapidly growing in the region, with promising interventions such as predictive analytics, automated detection of TB on CXRs, and personalised support. However, to maximise their benefit, emerging best practices and relevant frameworks for ethical use of data and algorithms, data representativeness, data quality, competency training for healthcare professionals, and health equity must be followed so that interventions can be safe, effective, ethical, and equitable in each context deployed

### Limitations

This scoping review has limitations. First, the broad heterogeneity in study populations, methodology, outcome measures, and data reporting precluded any quantitative synthesis of the comparative effectiveness of DHI interventions. Second, some articles are likely not captured despite the comprehensive search methods applied, due to the variability in aims, methodology, and outcome measures employed across DHI and respiratory health research. Third, it is likely that between the time the databases were searched and the time of this writing (a year difference), other data has emerged that present information on different DHIs for respiratory health due to the fast pace of technology development. Likewise, we searched both peer-reviewed and grey literature, hundreds of other interventions not reported in the literature likely exist in South and Southeast Asia, as a quick internet search reveals. Fourth, only studies in the English language were considered for inclusion. Fith, the specific geographical focus of this review made it impossible to include studies that may have had information about some of the relevant countries, but were global in scope and did not disaggregate results by country. This may have resulted in the exclusion of studies presenting more mature interventions that may be used across countries. Sixth, we ultimately excluded all COVID-19 studies due to their high number and lack of exclusive respiratory health focus. A COVID-19-only review could be undertaken to distil findings regarding DHIs for COVID-19 and assess whether those interventions are still in use and why. Finally, the mERA checklist was originally developed to report on mobile-based DHIs. We took the liberty of applying it to all interventions included in this study because it was deemed to be a tool fit for the purpose of this review, which sought to understand the completeness of DHI reporting. No study used predefined tools for evaluation and reporting; therefore, utilising the mERA checklist did not conflict with other reporting checklists employed by authors. These limitations notwithstanding, this scoping review offers results and critical themes that are in line with other comparable reviews [11-13], increasing the body of evidence while presenting novel results for respiratory health and the South and Southeast Asia regions specifically.

## CONCLUSIONS

The burden of respiratory disease in South and Southeast Asia is staggering, curtailing people’s lives and wellness. In this scoping review, we collated and synthesised information and knowledge on the current state of DHIs in the region, showcasing how such interventions are (or not) alleviating the respiratory health burden of disease and making relevant recommendations to researchers and practitioners. Our findings create a base of knowledge from which disrupting or sustaining innovations can be forged so that they are planned, deployed, scaled, and evaluated to have long-lasting positive impacts on the health and lives of the people we seek to serve.

## Additional material


Online Supplementary Document
